# Wisconsin State Dental Society

**Published:** 1871-09

**Authors:** 


### WISCONSIN STATE DENTAL SOCIETY.

This society met in La Crosse, on the 11th and 12th of July. There was not a large number present, but the active men of Wisconsin were there, and worked with energy and efficiency in the cause, and in behalf of their society. It was organized within the last two years, and for its age, is in a prosperous condition. The importance of State Associations, is just beginning to be understood and appreciated. State societies elicit the attention and interest of all the members of the profession in a State, to a greater extent, and more definitely, than any society of a more local character could do. Every enterprising, thorough going dentist, will feel himself called upon to enter and work with his State society, knowing that there he has a far larger sphere of usefulness than he could have in a small territory. The legitimate work of a State society will embrace all that can be done in a local society, and much more; it will or should in some sort take cognizance of the profession in the entire State, and labor for its well-being and advancement. It should make its influence felt by all of the profession in its domain.

Every State society should make a clear, unmistakable and strong expression upon the subject of professional education, one that would be regarded by all interested. It is proper and desirable for State societies to take such action as will give to the public a clear understanding of all things pertaining to the preservation of the teeth, and the capabilities of the dental profession to relieve from these difficulties, into which all to a greater or less extent sooner or later fall.

Another very important work of State societies, is the adoption of such measures, as shall discountenance, and if possible suppress quackery. All the definite means of progress tend in this direction; all are not alike prompt and decisive, however; something that shall be eminently vigorous and efficient is called for in the present state of affairs, and nothing would seem to promise more in this direction, than good, wholesome laws, that should encourage and sustain the good and upright, and discourage, prevent or punish, the dishonest and incompetent.

Another work in which every State society should engage, is the establishment of a normal institute, whose session of a few days each should be held at stated periods, and at places that would best accommodate those who are to avail themselves of its benefits. Such institutes should be conducted by some competent person or persons appointed by the State society, and for the benefit of practitioners who might choose to avail themselves of it. Such institutes would not operate in any sense against, nor take the place of dental colleges; but rather be co-operative with them, and aids to them. Now, these are some of the ways in which State societies may work great good for the profession. There are others of which we may speak hereafter. Some of these things the Wisconsin society has already taken up, and are acting upon, and others doubtless in due time will be taken up. We long to see the time when every State in the Union will have its dental society, each of which shall be instrumental in building up the art and science of our profession, and maintaining its honor. T.

